# The impact of the COVID-19 pandemic on the global dynamic spillover of financial market risk

**DOI:** 10.3389/fpubh.2022.963620

**Published:** 2022-08-02

**Authors:** Xiaoyu Tan, Shiqun Ma, Xuetong Wang, Chao Feng, Lijin Xiang

**Affiliations:** ^1^School of Finance, Zhongnan University of Economics and Law, Wuhan, China; ^2^School of Finance, Shandong University of Finance and Economics, Jinan, China

**Keywords:** financial market risk, COVID-19, dynamic spillover, TVP-VAR-Connectedness, CoVaR

## Abstract

The COVID-19 outbreak has greatly impacted the stability of the global financial markets. In the post-COVID-19 pandemic era, the risk contagion patterns of the global financial markets may change. This paper utilizes the conditional value-at-risk (ΔCoVaR) model to measure the risk level of the financial markets in various economies and uses the TVP-VAR-CONNECTEDNESS approach to construct a time-varying spillover index. Based on the dimensions of time and space, we explored the contagion path, contagion status, and contagion structure characteristics of global financial market risk before and during the COVID-19 pandemic. The results entail several conclusions. (i) The COVID-19 pandemic increased the spillover level of global financial market risk and the risk connectedness of financial markets in different countries. In addition, during the concentrated outbreak period of COVID-19, the risk spillover level in developing countries rose rapidly, while the financial risk spillover level in developed countries decreased significantly. (ii) The impact of the COVID-19 pandemic on the spillover of the global financial market risk is time-varying, and there is a strong correlation between the risk spillover level of the financial markets of the world and the severity of the COVID-19 pandemic. (iii) Due to the impact of the COVID-19 pandemic, Brazil, Canada, and Russia have become new risk spillover centers; in the post-COVID-19 pandemic era, China's spillover to developed countries has increased, and the financial influence of China has also gradually increased. In addition, the risk contagion capacity of financial markets among European countries is gradually converging. (iv) During the concentrated outbreak of the COVID-19 pandemic, the Americas were the main exporter of global financial market risk, while Europe played a role in risk absorption.

## Introduction

Economic globalization has become increasingly prominent since the 20th century due to technological breakthroughs, social development, and other aspects of progress. Despite the 2008 global financial crisis and the 2010 sovereign debt crisis, which devastated the global economy, the degree of financial integration continues to increase, and the increased connectedness among global financial markets is evident. In 2020 in particular, the outbreak and spread of COVID-19 significantly impacted the macroeconomic situations of all countries and global financial markets. As one of the most important financial submarkets, the stock market is the first to bear the brunt of major emergencies and is the key carrier of multilevel risk contagion. The US stock market crashed four times in March 2020. The Shanghai and Shenzhen stock market of China has accumulated more than 20% of the decline. Brazil, Japan, and other economies have also fallen sharply. Global stock market performance is sensitive, and the interaction among the stock markets is significant.

This diffusion and contagion phenomena of systemic financial risk among the international markets presents short-term, rapid, time-varying, and regional characteristics. Infection risk among markets, especially those due to stock market shocks following major emergencies (1), will exacerbate the downward pressure on the global economy and will also amplify the vulnerability of financial markets and catalyze the outbreak of financial crises (2, 3). The contagion effect of financial risk is defined by the significant increase in the connection intensity among markets during a crisis (4), and the linkage between financial markets is more prominent during the crisis (5). Given the continuous increase in financial integration, it is particularly necessary to study the impact of the COVID-19 pandemic on the dynamic connectedness of financial markets to prevent financial risk contagion.

In light of this situation, it is important to determine the characteristics of the cross-border contagion of financial market risk before and during the COVID-19 pandemic and the changes in global financial market risk due to the shock of the pandemic. Thus, based on the sensitivity of the stock market, this paper captures the cross-border spillover paths of financial market risk around the world, from both the longitudinal time dimension and the horizontal regional dimension, and deeply explores the microstructure and regional risk agglomeration of risk spillover.

The contributions of this paper to the existing literature are as follows. On the one hand, the TVP-VAR-Connectedness approach is introduced to deeply explore the cross-border contagion path, micro-transmission structure and its time-varying characteristics of global financial market risks before and during the impact of the COVID-19 epidemic, so as to clarify the ability of risk spillover, contagion patterns and contagion tendency of the sample countries in the post-COVID-19 epidemic era and provide policy basis and decision-making reference for the formulation and introduction of risk prevention policies of countries. On the other hand, this paper examines the spillover characteristics of global financial market risks in different continents based on the spatial dimension to clarify the regional contagion characteristics of financial market risks, so as to make up for the lack of existing literature and improve the pertinence of risk prevention policies.

The research shows that: First, the outbreak of the COVID-19 pandemic increased the risk spillover level of the global financial markets, increasing the density of the risk contagion network in the global financial markets and enhancing the risk connectedness among countries. In addition, due to the COVID-19 pandemic, the risk spillover levels of developing countries and developed countries have reversed. The risk spillover level of developing countries has risen rapidly, making developing countries the main exporters of financial market risk during the outbreak of the COVID-19 pandemic, while the financial risk spillover level of developed countries has decreased significantly, giving developed countries a role in risk absorption. Second, the impact of the COVID-19 pandemic on the risk spillover of the global financial markets is time-varying. In the post-COVID-19 pandemic era, there is a strong correlation between the risk spillover level of financial markets around the world and the severity of the COVID-19 pandemic. Third, due to the impact of the COVID-19 pandemic, Brazil, Canada, and Russia have become new risk spillover centers; in the post-COVID-19 pandemic era, the spillover of China to developed countries has gradually increased. In addition, the magnitude of net spillover contagion among European countries has decreased significantly, and the level of risk contagion among financial markets among countries has gradually converged. Lastly, during the concentrated outbreak period of the COVID-19 pandemic, the Americas were the main exporter of global financial market risk, while Europe played a role in risk absorption to some extent.

The reminder of this paper is organized as follows. Section Literature review presents the literature review. Section Methodology and data introduces our data and model applications. Section Empirical results shows the authentic proof analysis, and Section Conclusion finally presents our conclusions and policy implications.

## Literature review

With the accelerating process of economic globalization and financial integration, the economic connections among countries are becoming more and more closely, which provides conditions for the transnational transmission of financial risks. Therefore, the research on the risks connections among financial markets has become the focus of academic circles. Related research mainly takes financial sub-markets such as stock market (6–11), commodity market (11, 12), bond market (13, 14), foreign exchange market (15) and virtual currency market (16, 17) as the research objects, and conducts multi-directional identification and characterization analysis on the cross-market and cross-industry contagion characteristics of the financial risks under the impact of external emergencies, including the global financial crisis (GFC) and the European debt crisis (EDC).

At the beginning of 2020, the global outbreak of the COVID-19 epidemic has aroused widespread concern and attention in academia and practice. Cross-market and cross-industry contagion of financial risks under the impact of the COVID-19 epidemic has become a new research hotspot, such as financial risks contagion between commodity markets and stock markets (18–20), financial risks contagion among commodity markets (21, 22), and financial risks contagion between foreign exchange markets and commodity futures markets (23, 24). In addition, the cross-border contagion of financial risks under the impact of the COVID-19 epidemic is also an important area for many scholars to carry out research. Copula models, which are mostly used to capture the tail risk spillover effect among markets and describe the nonlinear correlation among financial markets, are the main research methods. For example, BenSaïda et al. (25) develops a tractable regime-switching version of the copula functions to model the risk connectedness of the stock market during turmoil and normal periods. However, this method is difficult to capture the time-varying characteristics of cross-border contagion of the financial risks, and the connectedness approach proposed by Diebold and Yilmaz (26, 27) has been recognized by the academic community in identifying the dynamic spillover correlation among the markets and among the countries. Therefore, the Diebold-Yilmaz spillover index is utilized by some scholars to study the dynamic cross-border financial risk contagion under the impact of the COVID-19 epidemic. For example, Li (28) uses Diebold-Yilmaz spillover index to study the time-varying volatility spillover effect of the stock markets in the US, Japan, Germany, the UK, France, Italy, Canada, China, India and Brazil. Choi (29) studied the dynamic correlation of stock market volatility in Northeast Asia, using the method of Diebold and Yilmaz (26). In addition, Akhtaruzzaman et al. (30) also used the approach of the spillover correlation of Diebold and Yilmaz (26) to study how financial risks in China and G7 countries were transmitted through financial and non-financial enterprises during the outbreak of the COVID-19 epidemic. However, it is worth noting that the construction of Diebold-Yilmaz spillover index is based on the rolling window VAR method, which has the problem of data loss and subjectivity in window size selection, while TVP-VAR-Connectedness approach (31) is an important means to solve this problem.

Additionally, there is no in-depth discussion on the cross-border contagion path of financial risks, microscopic contagion structure of financial risks and time-varying characteristics of the contagion path and the contagion structure among countries in the world. Moreover, the existing literature pays more attention to exploring the time-varying characteristics of financial risk contagion among countries, while ignoring the identification and research of spatial characteristics.

Based on this, this paper measures the financial risk level of sample countries, and uses the TVP-VAR-Connectedness approach to deeply explore the cross-border contagion path, microscopic structure of contagion and its time-varying characteristics of global financial market risks before and during the impact of the COVID-19 epidemic, in addition, we still captures the regional characteristics of financial risk contagion in order to make a more comprehensive explanation of financial risk contagion under the impact of the COVID-19 epidemic in the dual dimensions of time and space, which make up for the shortcomings of the existing literature and broadens the research breadth and depth of the existing literature.

## Methodology and data

### Methodology

#### ΔCoVaR model

To study the impact of the COVID-19 pandemic on the risk contagion level and multi-level spillover structure of the global financial markets, we firstly utilized the ΔCoVaR model (32) to obtain the VaR value of each country, that is, the financial market risk value of each country. We constructed the model as follows:

First, the quantile regression model of the financial market return sequence for a single economy can be expressed as follows:


(1) $$\begin{array}{l} R_{q}^{i}=\alpha^{i}+\varepsilon_{t}^{i}, \end{array}$$

where $R_{q}^{i}$ is the daily return sequences of the financial market of country *i*, and *q* is the quantile. When measuring risk, *q* is usually a small value (such as 1% and 5%). Our *q* is 5%, and we defined it as the state of risk in the financial market of a single country. Thus, when there is risk in a country's financial market, the sequence is as follows:


(2) $$\begin{array}{l} VaR_{5\%}^{i}={\hat{\alpha }}_{5\%}^{i} \end{array}$$

$VaR_{5\%}^{i}$ satisfies


(3) $$\begin{array}{l} P\left(X^{i}\leq VaR_{5\%}^{i}\right)=5\% \end{array}$$

where *X*^*i*^ represents the return of the financial market in country *i*, and $VaR_{5\%}^{i}$ represents the risk value of the financial market in country *i* under the *q* quantile.

#### The TVP-VAR model

After obtaining the financial market risk values, we used the TVP-VAR method proposed by Antonakakis and Gabauer (31) to construct the risk spillover index of financial markets around the world. This method introduces the forgetting factor proposed by Koop and Korobilis (33), which allows the variance to change through random-volatility Kalman filter estimation. This method therefore overcomes the subjective problem of selecting the rolling window size and further ensures the rationality of parameters and the integrity of data. In addition, it can still be used to check the dynamic correlation between low frequency and finite time-series data.

For a TVP-VAR model with *N* variables, each parameter is time-varying. Therefore, the VAR can be expressed by its vector moving average at any time. We subsequently estimated the spillover connectedness of the financial market risk among the sample countries based on the generalized impulse response function (GIRF) and the generalized forecast error variance decomposition (GFEVD) proposed by Koop et al. (34) and Pesaran and Shin (35) and employed by Diebold and Yilmaz (27).

The GIRFs ($\psi_{ij,t}^{g}\left(J\right)$) represent the change in financial market risk of country *j* after the spillover impact of the financial market risk of country *i* to country *j*. Based on the spillover shock capture method of Antonakakis and Gabauer (31), we computed the difference between a *j*-step-ahead forecast. The differences can be accounted for to measure the magnitude of the spillover shock of country *i*, which can be calculated with the following equations:


(4) $$\begin{array}{l} GIR_{t}\left(J,\delta_{j,t},F_{t-1}\right)=E(Y_{t+J}|\varepsilon_{j,t}\\ =\delta_{j,t},F_{t-1})-E\left(Y_{t+J}|F_{t-1}\right) \end{array}$$

(5) $$\begin{array}{l} \psi_{j,t}^{g}\left(J\right)=\frac{A_{J,t}S_{t}\varepsilon_{j,t}}{\sqrt{S_{jj,t}}}\frac{\delta_{j,t}}{\sqrt{S_{jj,t}}}\delta_{j,t}=\sqrt{S_{jj,t}} \end{array}$$

(6) $$\begin{array}{l} \psi_{j,t}^{g}\left(J\right)=S_{jj,t}^{-\frac{1}{2}}A_{J,t}S_{t}\varepsilon_{j,t}, \end{array}$$

where *Y*_*t*_ represents an *N* × 1 conditional volatility vector, and ε_*t*_ is an *N* × 1 dimensional error disturbance vector. *J* represents the forecast horizon; δ_*j, t*_ is the selection vector with 1 corresponding to the *jth* position, and 0 otherwise; *F*_*t*−1_ is the information set until *t* − 1; *S*_*t*_ is an *N* × *N* time-varying variance-covariance matrix; and $A_{t}={\left[A_{1,t},A_{2,t},\cdots ,A_{p,t}\right]}^{\prime }$.

#### The construction of the dynamic spillover connectedness index

Subsequently, we computed the GFEVD, which can be interpreted as the variance share one country has on others. These shares are then normalized so that each row sums up 1, meaning that all countries together explain 100% of the COVID-19 pandemic of country *i*. This is calculated as follows:


(7) $$\begin{array}{l} {\overset{\sim }{\phi }}_{ij,t}^{g}\left(J\right)=\frac{\sum_{t=1}^{J-1}\psi_{ij,t}^{2,g}}{\sum_{j=1}^{N}\sum_{t=1}^{J-1}\psi_{ij,t}^{2,g}} \end{array}$$

with $\sum_{j=1}^{N}{\overset{\sim }{\phi }}_{ij,t}^{g}\left(J\right)=1$, and $\sum_{i,j=1}^{N}{\overset{\sim }{\phi }}_{ij,t}^{g}\left(J\right)=N$. Through the GFEVD, the total spillover connectedness index (*TCI*) can be expressed as follows:


(8) $$\begin{array}{l} TCI_{t}^{\text{g}}\left(J\right)=\frac{\sum_{i,j=1,i\neq j}^{N}{\overset{\sim }{\phi }}_{ij,t}^{g}\left(J\right)}{\sum_{i,j=1}^{N}{\overset{\sim }{\phi }}_{ij,t}^{g}\left(J\right)}*100 \end{array}$$

(9) $$\begin{array}{l} =\frac{\sum_{i,j=1,i\neq j}^{N}{\overset{\sim }{\phi }}_{ij,t}^{g}\left(J\right)}{N}*100 \end{array}$$

Using the GFEVD, we also constructed the pairwise countries contagion index of the financial markets risk, which included the mean level ($C_{i\to j,t}^{g}\left(J\right)$) of contagion from country *i*to country *j* and is calculated as follows:


(10) $$\begin{array}{l} C_{i\to j,t}^{g}\left(J\right)=\frac{{\overset{\sim }{\phi }}_{ji,t}^{g}\left(J\right)}{\sum_{j=1}^{N}{\overset{\sim }{\phi }}_{ji,t}^{g}\left(J\right)}*100 \end{array}$$

The mean level ($C_{i\leftarrow j,t}^{g}\left(J\right)$) of contagion from country *j* to country *i* can be calculated as follows:


(11) $$\begin{array}{l} C_{i\leftarrow j,t}^{g}\left(J\right)=\frac{\overset{\sim }{\phi_{ij,t}^{g}}\left(J\right)}{\sum_{i=1}^{N}\overset{\sim }{\phi_{ij,t}^{g}}\left(J\right)}*100 \end{array}$$

We extracted Formulas (10) and (11) and defined the net pairwise countries contagion of the financial market risk from country *i* to country *j* as the contagion of the financial market risk from country *i* to country *j* minus the contagion of the financial market risk from country *j* to country *i*. This is calculated as follows:


(12) $$\begin{array}{l} C_{i,t}^{g}=C_{i\to j,t}^{g}\left(J\right)-C_{i\leftarrow j,t}^{g}\left(J\right). \end{array}$$

We calculated the magnitude of the contagion effect of the financial market risk between one country and other sample countries, such as the risk contagion transmitted by country *i*to other sample countries (*TO*_*it*_*Contagion*). This is expressed as follows:


(13) $$\begin{array}{l} TO_{it}=\overset{N}{\underset{j=1,j\neq i}{\sum }}C_{i\to j,t}^{g}\left(J\right) \end{array}$$

The risk contagion received by country *i* from other sample countries (*FROM*_*it*_ *Contagion*) is expressed as follows:


(14) $$\begin{array}{l} FROM_{it}=\overset{N}{\underset{i=1,j\neq i}{\sum }}C_{i\leftarrow j,t}^{g}\left(J\right). \end{array}$$

The net contagion denotes the difference between *TO*_*it*_*Contagion* and *FROM*_*it*_ *Contagion*, as shown in Formula (15):


(15) $$\begin{array}{l} NET_{it}=TO_{it}-FROM_{it}. \end{array}$$

### Data

To enhance the representativeness of the research conclusions and highlight the research significance of this paper, we selected the BRICS and G7 countries as research samples. The 12 sample countries include the major developing and developed countries in the world. These correspond to American countries, European countries, Asian countries, and African countries. The sum of the gross domestic product (GDP) of the sample countries in 2020 accounted for 67.65%^1^ of the total global GDP.

The COVID-19 pandemic impacted the volatility of the stock market most in all financial markets (36), and the risk contagion among stock markets has always been the focus of scholars' research. Therefore, we used the stock market of each country to refer to the financial market of the country, and we used the stock market return as the proxy variable of the financial market return. Additionally, we used the first-order logarithmic difference of the stock index of each country to represent the stock return of each country.

Table 1 shows the selection of stock price indices for sample countries, and the stock price index data for sample countries is from investing.com.

**Table 1:** Stock price index selection of sample countries.

**Country name**	**Stock price index**
China	Shanghai stock index
India	SandP CNX NIFTY index of India
Russia	MOEX Russia index
Brazil	IBOVESPA Stock index
South Africa	South Africa 40 index
The United States	SandP 500 index
The United Kingdom	UK 100 index
Germany	Germany DAX30 index
France	France CAC40 index
Italy	Italy 40 index
Japan	Nikkei 225 index
Canada	Toronto SandP_TSX composite index

*Stock price index data for sample countries come from Investing.com*.

To clarify the impact of the COVID-19 pandemic on the risk contagion path and the structural characteristics of the global financial market, the sample interval selected in this paper includes the pre-COVID-19 period and the COVID-19 period. We drew the subsamples selected in this paper from between October 12, 2017 and January 22, 2020 and between January 23, 2020 and May 20, 2022, respectively. We chose these periods based on the availability of sample data and to ensure the comparability of the estimation results of the two subsamples. We used the start date of the COVID-19 pandemic data published by Johns Hopkins University as the virus's outbreak date. We used the daily frequency information as the sample data. The two subsamples contain 559 and 560 daily data, respectively, and the full sample had a total of 13,428 daily frequency data. Table 2 presents the descriptive statistics for each variable.

**Table 2:** Description statistics.

**Variable**	**Observations**	**Mean**	**Std. dev**.	**Min**	**Max**	**Skewness**	**Kurtosis**
***Pre—COVID-19***							
China	559	0.008084	0.002366	0.005431	0.019863	1.868916	7.151405
India	559	0.006303	0.001967	0.004058	0.019008	2.54901	13.18086
Russia	559	0.006709	0.002456	0.004388	0.02718	4.367714	29.02445
Brazil	559	0.009341	0.001783	0.006671	0.017164	1.005048	3.992529
South Africa	559	0.00822	0.00191	0.005522	0.014336	0.8594661	3.114788
US	559	0.006536	0.003164	0.00373	0.021377	1.689028	5.529847
UK	559	0.006244	0.001559	0.004339	0.012517	1.640192	5.758704
France	559	0.006811	0.002338	0.0045	0.016427	1.563527	5.284173
Germany	559	0.007639	0.002194	0.004978	0.015647	1.226337	4.080824
Japan	559	0.008051	0.002491	0.005746	0.020856	1.982591	7.390921
Italy	559	0.007829	0.00227	0.005107	0.019799	1.499483	6.023216
Canada	559	0.00459	0.002	0.002704	0.013627	1.765956	
***During COVID-19*** **Variable**	**Observations**	**Mean**	**Std. dev**.	**Min**	**Max**	**Skewness**	**Kurtosis**
China	560	0.008195	0.002761	0.005433	0.027133	2.494082	11.54025
India	560	0.009518	0.00697	0.004001	0.056129	3.432736	17.07499
Russia	560	0.012733	0.015069	0.004738	0.136969	4.701141	30.26062
Brazil	560	0.012085	0.008411	0.006942	0.066649	4.188752	22.01396
South Africa	560	0.010298	0.00561	0.005781	0.045196	3.674201	18.58937
US	560	0.00937	0.007774	0.003877	0.069838	3.904768	22.4732
UK	560	0.008788	0.005184	0.004506	0.042661	3.302298	16.43826
France	560	0.010037	0.006824	0.004462	0.058292	3.44813	18.60456
Germany	560	0.010506	0.006679	0.004852	0.056913	3.26419	17.42234
Japan	560	0.009393	0.003485	0.005914	0.030484	2.326399	10.47743
Italy	560	0.010727	0.007987	0.005248	0.072495	4.211887	25.62474
Canada	560	0.007927	0.009418	0.002789	0.077833	4.75	28.42904

*The variables in the table are the returns of financial markets, this is, the returns of the stock markets. For example, “**China**” means the return of Chinese financial market*.

The sample data used in this paper are time-series data. Therefore, each sequence data must be tested for stability to ensure the accuracy of the estimation results. Thus, we conducted an augmented Dickey-Fuller unit root test on the return series of the financial markets of the sample countries during the pre-pandemic and pandemic periods. Table 3 shows that the data of financial market returns in various countries are stationary sequences.

**Table 3:** The results of Unit root tests of the each return sequences.

***Pre—COVID-19***			
***Variables***	***ADF test***	***Variables***	***ADF test***
*China-return*	−6.295^***^	*UK- return*	−6.945^***^
*India- return*	−6.070^***^	*France- return*	−6.776^***^
*Russia- return*	−7.577^***^	*Germany- return*	−6.859^***^
*Brazil- return*	−6.586^***^	*Japan- return*	−6.374^***^
*South Africa- return*	−7.865^***^	*Italy- return*	−6.402^***^
*US- return*	−7.199^***^	*Canada- return*	−6.025^***^
***During COVID-19 Variables***	***ADF test***	***Variables***	***ADF test***
*China- return*	−7.198^***^	*UK- return*	−6.654^***^
*India- return*	−6.127^***^	*France- return*	−6.535^***^
*Russia- return*	−6.724^***^	*Germany- return*	−6.157^***^
*Brazil- return*	−6.149^***^	*Japan- return*	−6.345^***^
*South Africa- return*	−6.265^***^	*Italy- return*	−6.164^***^
*US- return*	−5.964^***^	*Canada- return*	−6.305^***^

*The variables in the table are the returns of financial markets, this is, the returns of the stock markets. For example, “**China-Return**” means the return of Chinese financial market*.

*** *indicates the significance at the 1% level*.

## Empirical results

**Figure 1:**
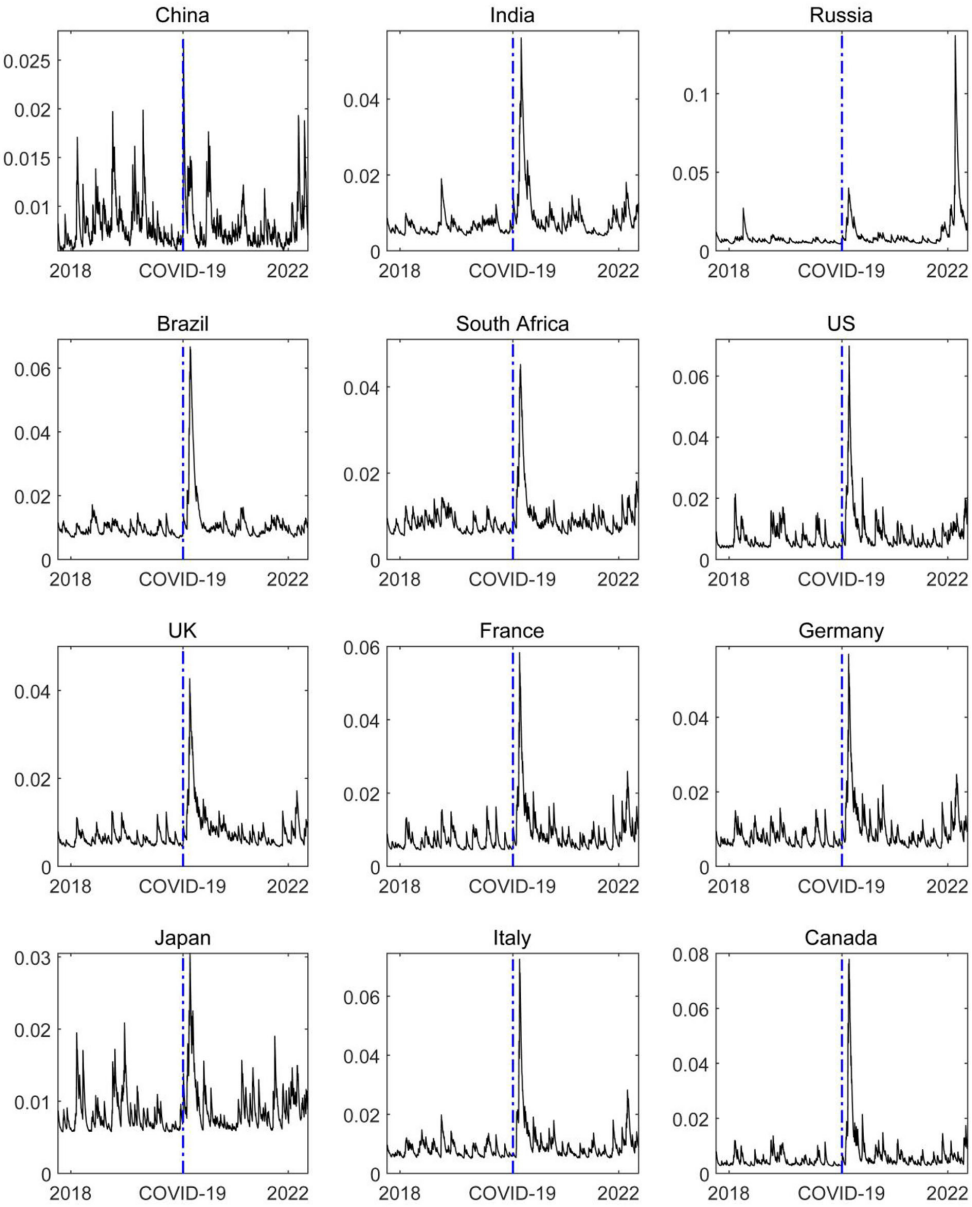
The trend of the magnitude of the financial market risks. The blue dash line represents the starting point of the COVID-19 epidemic, and the left and right sides of the blue dash line represent the dynamic change trend of the level of the financial market risks in the sample country.

The COVID-19 pandemic not only puts great pressure on global economic growth. It also has a negative impact on financial systems of countries that cannot be ignored, making the financial market risk soar (10). Therefore, we used the ΔCoVaR model to measure the level of financial market risk in various countries. The level of financial market risk and its trend are shown in Figure 1. Figure 1 shows that the COVID-19 pandemic has significantly intensified the financial market risk of various countries, which may be closely related to the risk aversion of investors and low risk preference under the impact of the COVID-19 pandemic. Therefore, the risk value of financial markets in various countries has risen to an absolute high level, especially at the initial stage of the event window period. In addition, it cannot be ignored that there are strong differences in the risk levels of financial markets across countries over time windows, showing obvious time-varying characteristics.

Accordingly, based on the time-varying perspective, this paper makes an in-depth study on the contagion path and the contagion structure characteristics of global financial market risk before and during the COVID-19 pandemic, and further clarifies the impact of the COVID-19 pandemic on the contagion of global financial market risk to provide corresponding theoretical support and decision-making reference for the formulation of the COVID-19 pandemic prevention and control policies.

### Time-varying analysis of risk contagion among global financial markets

To ensure the rationality and accuracy of the model estimation results, we employed the augmented Dickey-Fuller unit root test on the financial market risk level sequences measured above. Table 4 shows the test results. The financial market risk level sequences of the sample countries before and during the COVID-19 pandemic led us to reject the original hypothesis at the 5% level. Thus, the value-at-risk sequences of the financial markets in the sample countries have remained stable.

**Table 4:** The results of Unit root tests of the each risk sequences.

***Pre—COVID-19***			
***Variables***	***ADF test***	***Variables***	***ADF test***
*China-risk*	−4.544^***^	*UK- risk*	−4.983^***^
*India- risk*	−3.799^***^	*France- risk*	−4.982^***^
*Russia- risk*	−4.114^***^	*Germany- risk*	−4.823^***^
*Brazil- risk*	−4.151^***^	*Japan- risk*	−3.980^***^
*South Africa- risk*	−3.687^***^	*Italy- risk*	−4.972^***^
*US- risk*	−4.044^***^	*Canada- risk*	−4.315^***^
***During COVID-19 Variables***	***ADF test***	***Variables***	***ADF test***
*China- risk*	−4.213^***^	*UK- risk*	−3.488^***^
*India- risk*	−3.424^**^	*France- risk*	−3.704^***^
*Russia- risk*	−3.300^**^	*Germany- risk*	−3.629^***^
*Brazil- risk*	−3.812^***^	*Japan- risk*	−3.167^**^
*South Africa- risk*	−3.332^**^	*Italy- risk*	−3.877^***^
*US- risk*	−3.545^***^	*Canada- risk*	−4.116^***^

***“Country–Risk”** represents the financial market risk level sequences of the sample countries, for example, “**China-Risk**” represents the financial market risk level sequences of China*.

***and * *denote the significance levels of 1% and 5% respectively*.

To ensure the robustness of the results, we determined the lag order of the TVP-VAR model estimation process according to the Schwarz Bayesian information criterion because it selects more parsimonious models comparing to the Akaike information criterion (37), HQ (38, 39), and Akaike's final prediction error (37). The model can become overparameterized very quickly (40). Table 5 presents the selection of lag order in the model estimation process before and during the COVID-19 pandemic.

**Table 5:** The selection of lag order of TVP-VAR model.

**Periods**	**Lag order selection**
Pre—COVID-19	One
During COVID-19	One

*The lag order of the TVP-VAR model process is determined by the SBIC criteria*.

#### Time-varying total connectedness index (TCI)

**Figure 2:**
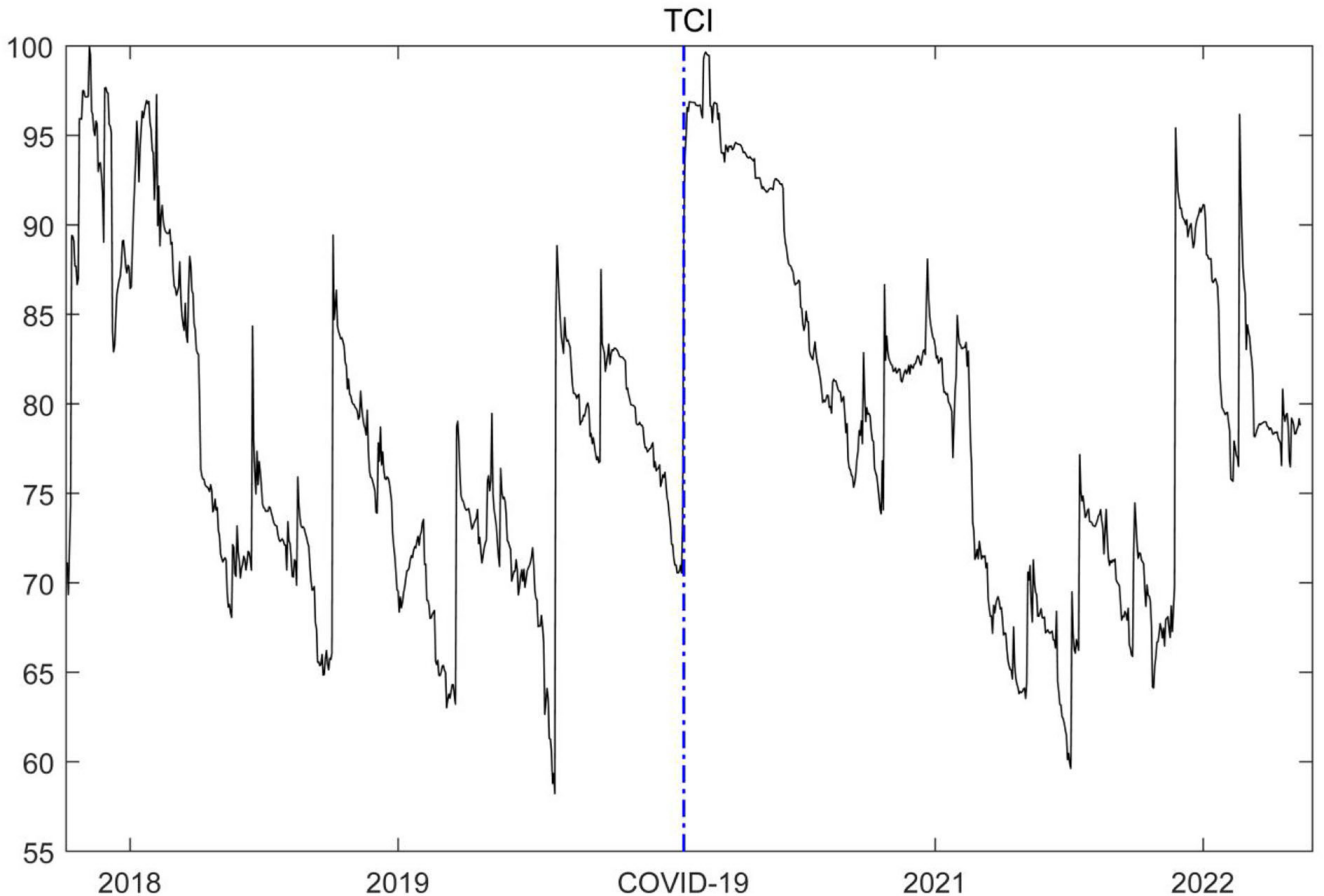
The time-varying total connectedness of the global financial market risks. The left side of the blue dash line represents the change trend of the total spillover level of the global financial market risks in the early stage of the COVID-19 epidemic, while the right side of the blue dash line represents the change trend of the total spillover level of the global financial market risks during the COVID-19 epidemic.

Figure 2 shows the change in the total spillover level of the global financial market risk before and during the COVID-19 pandemic. The figure shows there are two main peaks, one of which is in the early stage of the COVID-19 pandemic. The COVID-19 pandemic has significantly impacted the global financial market and macro economy, and the international capital market has experienced severe shocks. Especially in the early stage of the COVID-19 pandemic, the global supply chain was interrupted, investor panic intensified, and the systemic financial risk spread rapidly and cross-infected international markets. Therefore, the COVID-19 pandemic significantly increased the spillover level of the global financial market risk. Thus, the TCI rose rapidly to a high level in the short term after the COVID-19 pandemic, which is consistent with the research conclusions of Zhang et al. (19, 41), Cepoi (42), Benlagha and Omari (43) and Farid et al. (36), who found that risk contagion between stock markets increased remarkably during the health crisis outbreak.

Other peaks appeared at the end of 2017 and the beginning of 2018. The increase in the total spillover level of the global financial market risk is mainly related to Sino-US trade friction. Former President Trump authorized trade representatives to launch a “301 survey” on Chinese enterprises and on August 14, 2017. Consequently, trade friction between China and the US deepened, and unilateralism and trade protectionism increased, which increased the volatility of financial markets in various countries, as it influenced the microstructure and market information disclosure of financial markets in various countries, especially stock markets (44–46). Simultaneously, economic and trade ties and the level of financial openness have continuously improved in recent years. These are convenient channels for the cross-border contagion of financial risk in various countries. Therefore, the systemic financial risk contagion effect among financial markets has significantly increased. This finding proves that the model estimation results in this paper accurately capture the risk contagion effect among global financial markets affected by different major events and further verifies the robustness of the impact of the COVID-19 pandemic on the total spillover of global financial market risk.

#### From and to connectedness

**Figure 3:**
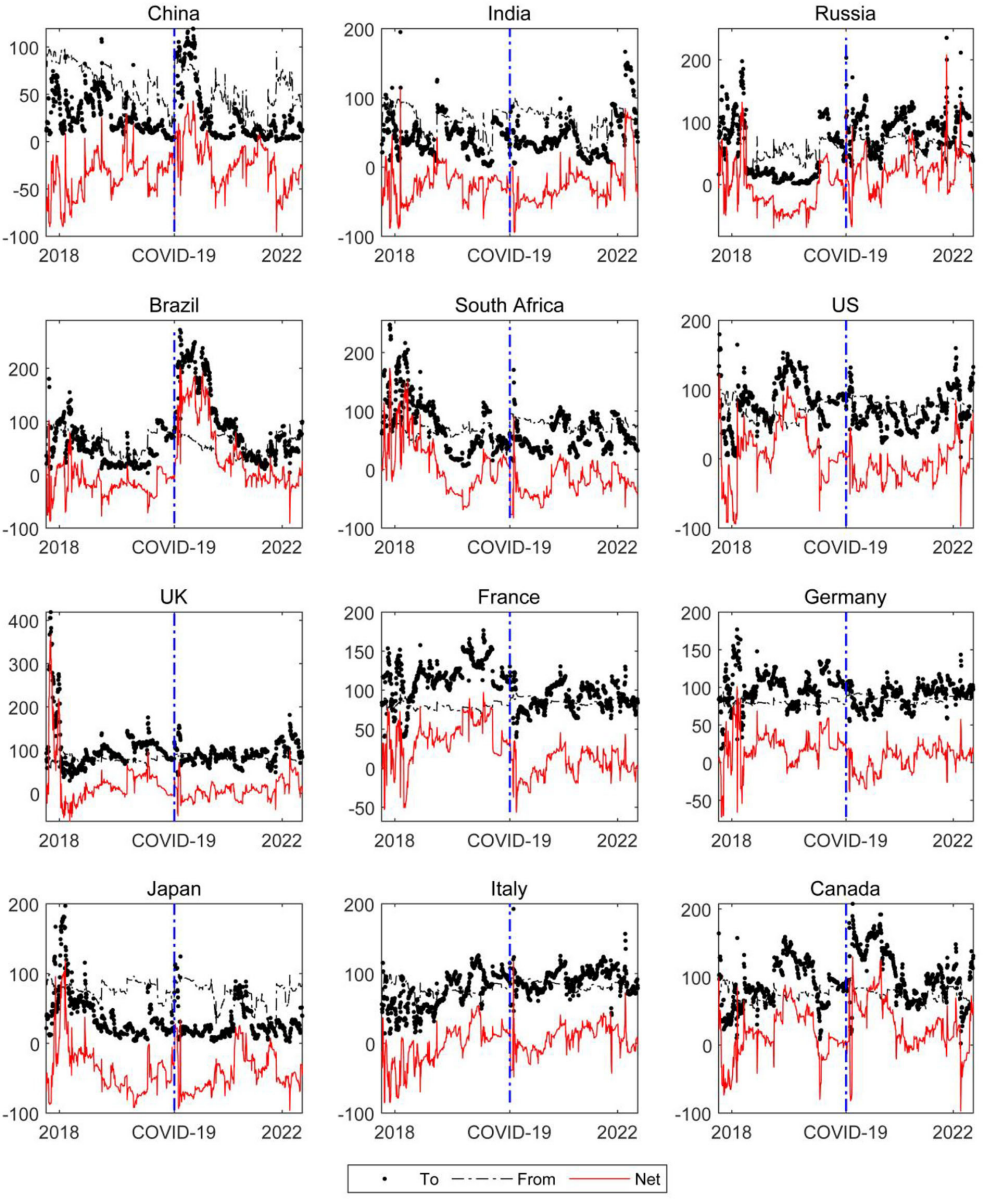
The time-varying directional connectedness of global financial market risk. The dotted line represents the contagion level of financial market risk of country *i* to other countries (*To*); the dash line indicates that the financial market risk level of country *i* is affected by the risk spillover of other countries (*From*); the red solid line represents the net spillover level of the financial market risk of country *i*, namely the difference between *To* and *From*.

The COVID-19 pandemic has had a heterogeneous impact on the financial markets of all countries in the world, resulting in obvious changes in the spillover level, spillover ability, and spillover status of financial market risk in various countries, and Akhtaruzzaman et al. (30) and Youssef et al. (47) found the same conclusion. However, it is worth noting that the existing literature does not conduct a detailed study on the structural characteristics, risk spillover paths, major global risk spillover points and regional characteristics of financial risk spillover among countries. This study just makes up for these deficiencies. Figure 3 shows that that, before the COVID-19 pandemic, the level of financial market risk in developing countries—including China, India, and Brazil—was seriously affected by the financial market risk of other countries. The shock of external risk made risk prevention and control challenging in developing countries. Developed countries create a strong financial risk contagion effect by virtue of their financial influence. For example, the US creates the main spillover of financial risk, and this spillover is also related to Sino-US trade frictions. The Sino-US trade dispute initiated by the US has caused pessimistic expectations for American investors, and its risk preference has also declined. Therefore, the US stock market, as the leader of the global stock market, has fluctuated greatly. This fluctuation has brought severe negative impacts on the capital markets of all countries. Accordingly, there is a high spillover level of the financial market risk in the early stage of the COVID-19 pandemic in US.

However, after the outbreak of the COVID-19 pandemic, the risk spillover levels of developed and developing countries have changed significantly. The spillover level of the financial risk in developing countries increased significantly with the outbreak of the COVID-19 pandemic. For example, the risk spillover of financial markets in China, Russia, and Brazil increased significantly, and the risk spillover level increased rapidly to a relatively high level at the beginning of the COVID-19 pandemic. It is worth noting that the dynamic trend of risk spillover level in developed countries shows a “U-shaped” trough at the beginning of the COVID-19 pandemic, indicating that the net contagion of financial risk in developed countries decreased after the outbreak of the COVID-19 pandemic. This entails these countries have a strong risk-absorption capacity. The important reason for the reversal in financial risk contagion between developed and developing countries due to the COVID-19 pandemic may be that there is a great difference in the development of the financial markets between the two groups of countries. Thus, there is significant heterogeneity between developed and developing countries abilities to control their own financial risk. For example, the maturity of financial markets in developing countries and the proportion of institutional investors in the market are both relatively low. The market lacks the ability to share risk, and the capital allocation function of the market is also be jeopardized. Therefore, with major emergencies, it is difficult for developing countries to effectively digest their own financial risk, so the risk spillover effect is enhanced. However, the basic system and infrastructure of financial markets in developed countries are relatively complete. When there is high global economic uncertainty, developed countries often play a safe haven role. A large number of capital flows into developed countries, such as the US, the UK, and Germany, for the purpose of risk aversion. The level of financial market risk contagion in countries such as the US, the UK, and Germany shows a downward trend during the early outbreak of the COVID-19 pandemic.

Furthermore, the risk spillover level of financial markets around the world in the post-COVID-19 pandemic era is related to the severity of the COVID-19 pandemic to some extent, as the spillover level shows strong time-varying characteristics. As the time window expands, the impact of the COVID-19 pandemic on the net-risk spillover ability of financial markets in different countries gradually weakens, which is mainly related to the increase of the COVID-19 epidemic prevention and control and the gradual dissipation of the panic of investors (48, 49). However, with frequent outbreaks, the level of risk spillover in different countries is still changing frequently, which is similar to what is seen at the beginning of the COVID-19 pandemic. For example, the COVID-19 pandemic entered its fourth wave in December 2021, and the financial risk spillover level of Russia, India, and other countries reached a high level again.

It is worth noting that, under the impact of the COVID-19 pandemic, the risk spillover center of the global financial markets has shifted, and Brazil, Canada, and Russia have become the main spillover countries of financial market risk in the post-COVID-19 pandemic era.

#### Net pairwise directional connectedness

Previous results have captured and identified the net spillover level and spillover status of financial market risk around the world before and during the COVID-19 pandemic. This paper further measures and depicts the dynamic changes of the point-to-point contagion magnitude of financial market risk among the countries around the world before and during the COVID-19 pandemic to clarify the impact of the COVID-19 pandemic, the contagion path, and characteristics of the global financial market risk in the post-COVID-19 pandemic era.

**Figure 4:**
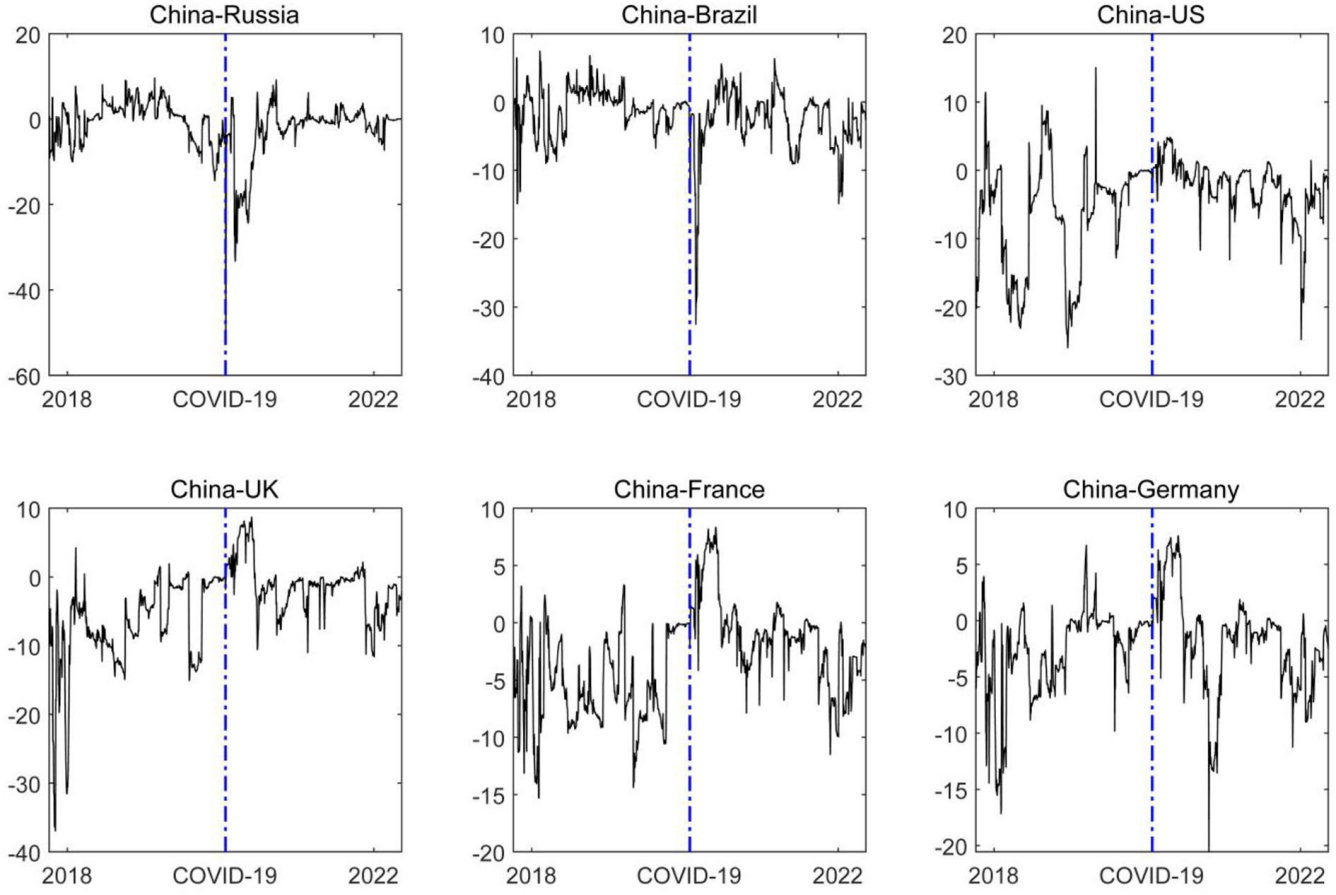
The dynamic net pairwise spillover effect the financial market risks in China. The blue dash line represents the starting point of the COVID-19 epidemic. In the figure, the left country is the risk spillover country, and the right country is the risk receiver. The solid line in the figure is the net spillover level between the two countries.

Figure 4 shows the dynamic changes in the level of net spillover between China and other countries. We can find that China, as the world's second largest economy, has a relatively strong financial impact in developing countries. For example, before the COVID-19 pandemic, the risk arising from the volatility of the financial market in China had a strong positive spillover effect on Russia and Brazil. That is, the development of the financial market of China is a sort of guiding force for other developing countries. However, the financial risk in developed countries such as the US and the UK have a strong spillover impact on China, indicating that the maturity of the financial markets in China can be further improved. The outbreak of the global COVID-19 pandemic has exposed all economies to common risks. Such major emergencies pose a huge challenge to the financial stability of all countries, which is also a test of the risk tolerance and absorption capacity of the financial markets of all countries. Among them, China shows a certain degree of risk-absorption capacity in developing countries. In the early stage of the COVID-19 pandemic, China still had a significant spillover impact on other developed countries, while over time, the magnitude of risk contagion between China and other developed countries gradually returned to the state before the COVID-19 pandemic. It is worth noting that the spillover impact of the US, the UK, and other developed countries on China is significantly reduced, which indicates that the financial influence of China, especially on developed countries has increased in the post-COVID-19 pandemic era, and the strong economic resilience and institutional superiority of China under the continuous impact of the COVID-19 epidemic are important reasons for ensuring the gradual enhancement of China's economic and financial influence and the steady improvement of international status. For example, China's economy has taken the lead in recovery under the influence of the COVID-19 epidemic^2^, and has been widely recognized by the international community.

**Figure 5:**
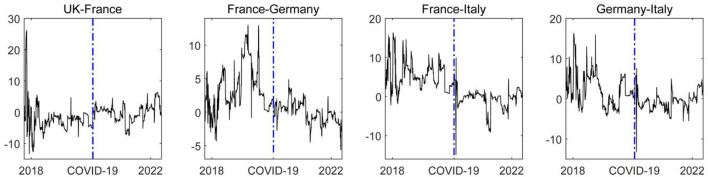
The dynamic net pairwise spillover effect of the financial market risks among European countries. The blue dash line represents the starting point of the COVID-19 epidemic. In the figure, the left country is the risk spillover country, and the right country is the risk receiver. The solid line in the figure is the net spillover level between the two countries.

Results regarding developed European countries are shown in Figure 5. Therefore, in the post-COVID-19 pandemic era, the magnitude of net financial market risk spillover among European countries has decreased significantly, and the ability of the financial market risk contagion among the countries has gradually converged. The COVID-19 pandemic has seriously impacted the economic and financial development of these countries through common risk exposure. Accordingly, the net spillover level of financial market risk among the countries tends to zero. In addition, the spillover level of financial market risk among European countries still fluctuates around zero due to the differences in the level of development of the financial market infrastructure and basic systems across European countries.

**Figure 6:**
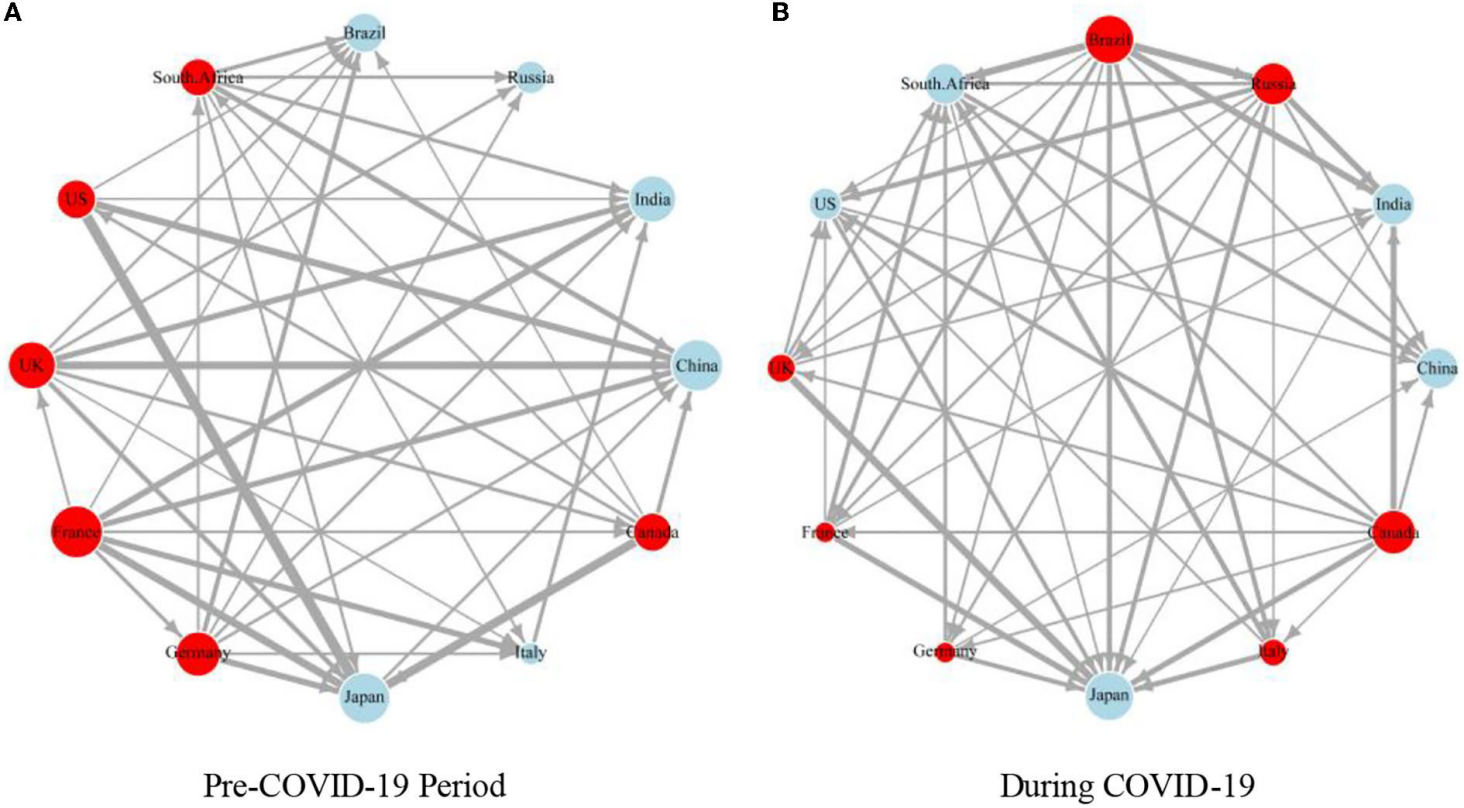
The cross-border spillover weighted network of global financial market risks before and during the COVID-19 epidemic. **(A)** Pre-COVID-19 Period and **(B)** During COVID-19. The red (blue) node is the financial market risk exporter (receiver). In this paper, the sum of net spillover (NET) of nodes is used to weight each node, and the size of nodes is used to represent the spillover level of each country and the degree of influence by external risks. At the same time, this paper takes the net spillover level among countries as the edge weight, and the risk net spillover level among countries is expressed by the width of the edge.

To clearly depict the risk contagion path of financial markets in various countries, we made a visual analysis of the global financial risk contagion path. Figure 6A shows the results. The figure shows that, after the outbreak of the COVID-19 pandemic, the density of the risk spillover network in the global financial market increased significantly, and the risk connectivity among countries increased, indicating that the global risk contagion has intensified in the post-COVID-19 pandemic era and that the prevention and control of the transnational contagion of financial risk is necessary for all countries. In addition, due to the COVID-19 pandemic, Brazil, Canada, and Russia have become the new financial risk spillover centers, which is consistent with the above conclusion and confirms the robustness of the above conclusion.

It is worth noting that the size of European nodes is gradually similar, which shows that the spillover capacity of the UK, France, Germany, and Italy is similar, and the transnational contagion of financial market risk may possess regional characteristics.

### Regional analysis of risk contagion among global financial markets

Based on a time-varying analysis, this paper further explores the contagion of global financial market risk on a regional dimension. Figure 6B shows that the COVID-19 pandemic has significantly increased the spillover ability of the financial market risk in the Americas. Therefore, in the early stage of the COVID-19 pandemic, namely, during the concentrated outbreak period, the Americas are the main exporters of the global financial market risk. The reason for this occurrence may be that there were three global centers of the COVID-19 pandemic in the Americas: Canada, Brazil, and the US. The interactive contagion of the COVID-19 pandemic has doubled the difficulty of preventing COVID-19 spread in various countries and put the financial markets in a state of turbulence (50). For example, the stock markets of various countries have triggered the melting down one after another, which has intensified the financial market risk in the Americas. Thus, the net contagion level of the financial market risk in the Americas surged in the short term. Over time, the impact of the COVID-19 pandemic on investors gradually weakened, market panic disappeared, and the level of risk spillover in the Americas declined. However, it still increases periodically with new waves of the pandemic. Overall, the level of the Americas' risk spillover in the post-COVID-19 pandemic era is relatively high.

It is also worth noting that Europe played a role in risk absorption to some extent during the concentrated outbreak period of the COVID-19 pandemic. For example, since the outbreak of the COVID-19 pandemic in February 2020, the net spillover level of the financial risk in Europe dropped rapidly below the zero-scale line, thus receiving the spillover impact of external risk. Even with the decline in the severity of the global COVID-19 pandemic, European financial markets still show a strong influence, and the level of the risk spillover has risen to a high level again. Accordingly, Europe is an important exporter of global financial risk when the COVID-19 pandemic is stable.

## Conclusion

This paper used the ΔCoVaR model to measure the risk level of the financial markets in various economies and the TVP-VAR-CONNECTEDNESS approach to construct a time-varying spillover index. Based on the dimensions of time and space, we explored the contagion path, contagion status, and contagion structure characteristics of global financial market risk before and during the COVID-19 pandemic. The main conclusions of this paper are as follows.

First, the outbreak of the COVID-19 pandemic increased the risk spillover level of the global financial markets, increasing the density of the risk contagion network in the global financial markets and enhancing the risk connectedness among countries. Therefore, in the post-COVID-19 pandemic era, it is particularly necessary to prevent and control the transnational transmission of financial risk. In addition, due to the COVID-19 pandemic, the risk spillover levels of developing countries and developed countries have reversed. The risk spillover level of developing countries has risen rapidly, making developing countries the main exporters of financial market risk during the outbreak of the COVID-19 pandemic, while the financial risk spillover level of developed countries has decreased significantly, giving developed countries a role in risk absorption.

Second, the impact of the COVID-19 pandemic on the risk spillover of the global financial markets is time-varying. With the expansion of the time window, the impact of the COVID-19 pandemic on the net-risk spillover impact of financial markets in various countries gradually weakens. In the post-COVID-19 pandemic era, there is a strong correlation between the risk spillover level of financial markets around the world and the severity of the COVID-19 pandemic.

Third, due to the impact of the COVID-19 pandemic, the risk spillover centers of global financial markets have shifted, and Brazil, Canada, and Russia have become new risk spillover centers; in the post-COVID-19 pandemic era, the spillover of China to developed countries has gradually increased, and the financial influence of China has increased. In addition, the magnitude of net spillover contagion among European countries has decreased significantly, and the level of risk contagion among financial markets among countries has gradually converged.

Lastly, during the concentrated outbreak period of the COVID-19 pandemic, the Americas were the main exporter of global financial market risk, while Europe played a role in risk absorption to some extent.

Some policy implications can be drawn from the above conclusions. First, the governments of developing countries should coordinate the prevention and control of the pandemic and risk supervision and establish a dynamic financial risk early warning mechanism in combination with changes in domestic pandemic prevention measures. And at the micro level, the government should analyze and explain the emergency cases and improve the market information disclosure system to avoid group irrational behavior. Second, considering the close and time-varying correlation between the severity of the pandemic and financial risk spillover, governments should prevent the possibility of a worsening or re-emerging pandemic and combine short-term rescue with long-term support policy, detailed analysis of infection channel diversification under the background of epidemic. Third, in view of the changes in the global risk spillover pattern caused by the pandemic shock, international organizations should consider establishing coordinated international disposal mechanisms and profit and loss-sharing mechanisms led by central banks for integrated regulation and information exchange. For example, regarding the gradual convergence of the risk contagion capabilities within the euro zone, governments and international organizations could consider multilateral and regional monetary policy coordination, strengthen the monitoring of cross-border capital flows, and weaken the intensity of risk-hedging attacks with risk diversification. Fourth, considering the geographical dimension of risk contagion, especially the role of risk spillover changes in Europe, governments should actively guide investors' overseas investment tendencies and risk expectations, and give full play to the regional risk absorption efficiency and correction ability to achieve mutual risk prevention in the region.

Although the research conclusion of this paper makes up for the academic research vacancy to some extent, there are still some limitations in the research content, for example, this paper only takes the major global economies as sample countries, so the number of sample countries is still relatively small. Future longitudinal studies are needed to expand sample size and carry out all-round research on the contagion of the financial risks in the post-epidemic era.

## Data availability statement

The original contributions presented in the study are included in the article/supplementary material, further inquiries can be directed to the corresponding author/s.

## Author contributions

XT: conceptualization, validation, writing–original draft, supervision, and funding acquisition. SM: methodology and writing—review and editing. XW: writing—review and editing and visualization. CF: software, resources, and data curation. LX: software, data curation, project administration, and visualization. All authors contributed to the article and approved the submitted version.

## Funding

We acknowledge the financial support from Youth Foundation of Humanities and Social Sciences of the Ministry of Education in China (20YJC790122). All errors remain our own.

## Conflict of interest

The authors declare that the research was conducted in the absence of any commercial or financial relationships that could be construed as a potential conflict of interest.

## Publisher's note

All claims expressed in this article are solely those of the authors and do not necessarily represent those of their affiliated organizations, or those of the publisher, the editors and the reviewers. Any product that may be evaluated in this article, or claim that may be made by its manufacturer, is not guaranteed or endorsed by the publisher.

## Footnotes

^1^Data Source : the World Bank WDI Database.

^2^Data Source: Economic database of the Federal Reserve Bank of St. Louis (https://fred.stlouisfed.org).
